# The Effect of Interpersonal Counseling for Subthreshold Depression in Undergraduates: An Exploratory Randomized Controlled Trial

**DOI:** 10.1155/2018/4201897

**Published:** 2018-02-22

**Authors:** Ami Yamamoto, Emi Tsujimoto, Reiko Taketani, Noa Tsujii, Osamu Shirakawa, Hisae Ono

**Affiliations:** ^1^Department of Psychological Science, Graduate School of Humanities, Kwansei Gakuin University, 1-155 Uegahara Ichibancho, Nishinomiya, Hyogo 662-8501, Japan; ^2^Department of Neuropsychiatry, Kindai University Faculty of Medicine, 377-2 Ohno-Higashi, Osakasayama, Japan

## Abstract

**Background:**

Subthreshold depression and poor stress coping strategies are major public health problems among undergraduates. Interpersonal counseling (IPC) is a brief structured psychological intervention originally designed for use in primary care to treat depressive patients whose symptoms arose from current life stress.

**Objectives:**

This study examined the efficacy of IPC in treating subthreshold depression and coping strategies among undergraduates in school counseling.

**Materials and Methods:**

We carried out an exploratory randomized controlled trial comparing the efficacy of IPC with counseling as usual (CAU). Participants were 31 undergraduates exhibiting depression without a psychiatric diagnosis.

**Results:**

The Zung Self-Rating Depression Scale total score decreased significantly in the IPC group (*n* = 15; *Z* = −2.675, *p* = .007), but not in the CAU group (*n* = 16). The task-oriented coping score of the Coping Inventory for Stressful Situations showed a tendency towards a greater increase in the IPC group than in the CAU group (*t* = 1.919, df = 29, *p* = .065).

**Conclusions:**

The IPC might be more useful for student counseling because it can teach realistic coping methods and reduce depressive symptoms in a short period. Further studies using more participants are required.

## 1. Introduction

Recently, researchers have pointed out a high prevalence of depression among Japanese undergraduates [1, 2]. Several explanations have been posited; undergraduates are confronted with numerous problems inherent to adolescence and emerging adulthood, such as the establishment of their identity, achieving independence from parents, entering an unfamiliar environment at college, striving for good academic performance, and selecting an occupation after graduation. In addition to dealing with these problems peculiar to undergraduates, they are often unaware of the realistic coping strategies for addressing these problems. They often use inadequate coping strategies, which can make matters worse. Particularly, some studies have reported that strong emotional and avoidance coping strategies were positively related to depression among undergraduates [3–5].

The number of undergraduates seeking school counseling for their depression has been increasing. Their depression is usually mild and does not meet the diagnostic criteria for clinical depression and does not require medical treatment at a hospital [6, 7]. Hence, they ask for school counseling from their university. However, it can be difficult to support depressive undergraduates through student counseling, which, in Japan, tends to focus on self-resolution and growth through psychological conflict; furthermore, it often takes a long time to see results [8].

Interpersonal counseling (IPC) is a brief, structured psychological intervention derived from interpersonal psychotherapy. IPC was originally designed for use in primary care by a variety of disciplines, including primary care physicians, nurses, and counselors, in order to treat depressive patients whose symptoms arose from current life stress [9, 10]. In principle, IPC comprises three 50-minute sessions focusing on patients' current interpersonal problems and social functioning in currently stressful areas. The aim of IPC is not to change the client's personality, but rather to help the client escape from temporary problems using appropriate coping strategies [11, 12]. Considering the characteristics of IPC, it could be highly practical and effective if integrated into student counseling for undergraduates exhibiting depression and poor stress coping strategies. However, at present, there are no studies on the effectiveness of IPC among Japanese undergraduates.

We therefore carried out an exploratory randomized controlled trial (RCT) in which we compared the efficacy of IPC with that of counseling as usual (CAU) for undergraduates with depression. The primary objective of the study was to determine whether IPC was more effective than CAU for managing the symptoms of depression, while the secondary objective was to determine whether IPC had a stronger positive effect on coping strategies. We hypothesized that IPC would lead to a greater reduction in depression and a greater change in coping strategies than CAU would.

## 2. Method

### 2.1. Design and Participants

This was an exploratory RCT using a single-blind, crossover design to compare IPC and CAU; it was conducted from 2013 to 2015. This study was approved by the Kwansei Gakuin University Regulations for Research with Human Participants and conforms to the tenets of the declaration of Helsinki.

**Figure 1:**
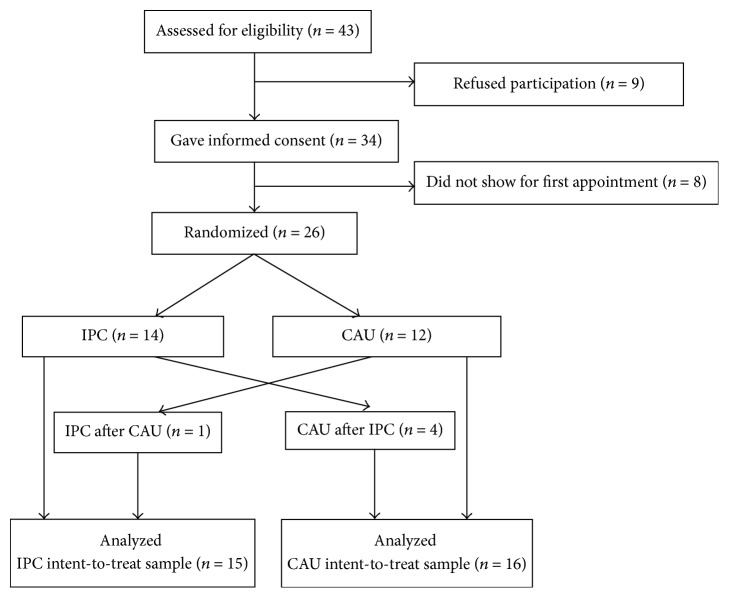
Recruitment and retention of participants. CAU is counseling as usual; IPC is interpersonal counseling.

We recruited participants in class from Kwansei Gakuin University. The inclusion criteria were being undergraduates aged 20–39 years who complained of subjective depression, while the exclusion criteria were having a mental disorder confirmed using the Mini-International Neuropsychiatric Interview [13, 14]. Eligible undergraduates (*n* = 43) who met all inclusion and exclusion criteria signed written informed consent forms after receiving an explanation of the study procedures and an opportunity to ask questions. After their consent was obtained, participants were randomly assigned to the IPC (*n* = 14) or CAU (*n* = 12) groups, alternating by gender. Once participants had completed their respective counseling sessions, those who wanted to receive the other type of counseling and who still met the inclusion criteria underwent a washout period of at least one week before undergoing the other counseling (IPC: *n* = 1, CAU: *n* = 4). Through such a study design, we planned to maximize the amount of data obtained from a small sample size. We also made sure that participants could receive both forms of counseling as part of our ethical considerations. Unexpectedly, rather few wanted to receive the other type of counseling. All participants who received the counseling (IPC: *n* = 15, CAU: *n* = 16) completed all sessions.  Figure 1 illustrates the study design.

### 2.2. Procedure

Prior to randomization, all participants responded to a questionnaire on their demographic information, including age and gender, as well as free description about their problems and subjective depression. Furthermore, they underwent the MINI interview with a psychiatrist. Once participants had been assigned to the IPC or CAU group, they completed self-rated scales as a precounseling assessment. After receiving the counseling (postcounseling), participants again completed the self-rated scales.

### 2.3. Assessments

Participants' total score on the Japanese version of the Zung Self-Rating Depression Scale (SDS) [15, 16] was used as an indicator of depression. The SDS comprises 20 items assessing a depressive state, each rated on a 4-point response scale (1 is* a little of the time*, 4 is* most of the time*). The total score on the SDS ranges from 20 to 80. Using the traditional cut-off score for the SDS, we designated all respondents who scored over 39 as having depression. The SDS has been shown to have good split-half reliability and content validity [16]. The SDS was administered before and after counseling.

The Japanese version of the Coping Inventory for Stressful Situations (CISS) [17, 18] was used to assess the coping strategies of the three-factor model. The CISS comprises 48 items asking how often they engage in various activities when they encounter a stressful situation. Each item is rated on a 5-point scale (1 is* not at all*, 4 is* very much*), which together assess three coping strategies: task-oriented coping, emotion-oriented coping, and avoidance-oriented coping. Task-oriented coping refers to taking an active problem-solving approach to stressful situations; emotion-oriented coping refers to confronting stressful situations with strong emotional responses; and avoidance-oriented coping refers to simple avoidance of dealing with the problem at hand. The scores for each coping strategy range from 16 to 80, with higher scores indicating a greater tendency to use the coping strategy in question. The CISS has good internal consistency and test-retest reliability [18]. The CISS was administered before and after counseling.

### 2.4. Intervention

IPC was performed in accordance with the official manual [11]. The IPC intervention comprised three 50-minute sessions that focused on participants' current problems. Through these sessions, participants were helped to identify effective strategies for managing their interpersonal problems. The CAU intervention also comprised three 50-minute sessions. The CAU technique was counselee-centered and supportive and is the standard for use in student counseling in Japan. Counselors were graduate psychology students. To ensure that the intervention was consistently delivered, counselors attended a 1-day teaching seminar on interpersonal therapy technique, and they performed a number of simulated IPC and CAU sessions before participating in the study. They all received weekly group supervision by an experienced psychiatrist during the study to ensure the quality of the counseling.

### 2.5. Outcomes and Statistical Analyses

The primary outcome was pre-post change in SDS total score within the two groups, as well as how these changes differed between the groups. The secondary outcome was the pre-post change in task-oriented coping, emotion-oriented coping, and avoidance-oriented coping scores within the counseling groups, and how these changes differed between groups.

We performed all analyses on an intent-to-treat sample (IPC: *n* = 15, CAU: *n* = 16). Initially, we confirmed whether each scale score followed a normal distribution using the Shapiro-Wilk test. A *t*-test was performed for variables that showed a normal distribution, while the Mann–Whitney *U* test (for between-groups comparisons) and Wilcoxon signed-rank test (for within-groups comparisons) were performed for variables without a normal distribution. Differences in categorical variables were tested via chi-square tests. All statistical analyses were performed using SPSS Statistics 23. We set a statistical significance level at 5% (two-tailed).

## 3. Results

### 3.1. Characteristics of Participants

Table 1 shows the characteristics of the 31 participants for whom data were analyzed (6 men, 25 women; 20.7 ± 1.1 years old). We observed no significant differences in demographic characteristics between the IPC and CAU groups. For problems among all participants, problems with interpersonal relationships were reported most frequently (48%), followed by problems with studying (26%) and problems with future career (16%).

### 3.2. Outcomes

Table 2 shows the comparison of primary and secondary outcomes within and between the IPC and CAU groups.

#### 3.2.1. Primary Outcome

The SDS total score decreased significantly after counseling in the IPC group (*Z* = − 2.675, *p* = .007*, Wilcoxon signed-rank test*), but not in the CAU group (*t* = − .068, df = 15, *p* = .947*, t-test*). Furthermore, the decrease in the SDS total score was significantly larger in the IPC group than in the CAU group (*t* = − 2.300, df = 29, *p* = .029,* t-test)*.

#### 3.2.2. Secondary Outcome

The task-oriented coping score of the CISS did not show a significant change in either the IPC group or the CAU group. However, the IPC group score showed a tendency towards a larger increase than that in the CAU group (*t* = 1.919, df = 29, *p* = .065, *t-test*). We also detected no significant changes in emotion-oriented or avoidance-oriented coping scores within the IPC group, or within the CAU group.

## 4. Discussion

This is the first RCT to investigate the efficacy of IPC for subthreshold depression and improving coping strategies of undergraduates with subthreshold depression. The primary finding of this study was that the SDS total score decreased more in the IPC group than in the CAU group. The secondary finding was the change in task-oriented coping score on the CISS showed a tendency towards a significant difference between the IPC and CAU groups.

Regarding the level of depressive symptoms in participants, we found that the mean total SDS score of all participants was in line with that reported in previous studies of Japanese undergraduates [19, 20]. As for the coping strategies, all of the mean scores fell into the average range based on a Japanese reference group [21]. Thus, it would seem that our participants were average undergraduates in terms of depression and coping strategies, despite their complaints of depression and problems. Moreover, the finding that they had more problems related to their relationships with others than with studying or their future career might indicate that undergraduates have suffered from problems with their interpersonal relations more than mere student-specific problems. This result suggests that IPC, which focuses on clients' current interpersonal problems and social functioning [12], could be an appropriate and effective counseling method for depression in undergraduates.

The hypothesis that the IPC intervention would help alleviate depression more than CAU would was supported. Importantly, IPC was effective in reducing depression despite being given for only three 50-minute sessions. IPC appears to be useful for teaching realistic communication methods in a short period to undergraduates. A possible mechanism by which IPC decreased depression was that it taught undergraduates to use various communication methods to help resolve the problems causing them depression, thus leading to an improvement. On the other hand, the CAU likely could not produce an effect over three 50-minute sessions because it generally strives to encourage self-resolution and growth through psychological conflict, which takes much longer to achieve.

We found no support for the hypothesis that IPC would lead to a greater change in coping strategies. However, there was a tendency for task-oriented coping strategy to increase in the IPC group. IPC teaches participants realistic communication methods to help resolve stressful events; therefore, IPC might improve use of the task-oriented coping, which involves taking an active problem-solving approach to dealing with problems.

This study has some limitations. First, this was an exploratory trial using a crossover design and the correlations between the improvement in depression and the change in coping strategies were not directly studied. Further studies with larger samples are needed. Second, we employed self-report measures for all variables, which opens up our results to the potential for social desirability bias, despite the fact that all participants were informed that their responses would remain anonymous. Third, there might have been some selection bias operating because participation was voluntary; in other words, it is possible that only those with high expectations of counseling took part in the study. Finally, we only investigated the effect after completing counseling. Studies aiming to determine long-term effects are required.

## 5. Conclusions

This exploratory RCT study found the possibility that IPC showed a greater reduction in depression compared to usual counseling in undergraduates with subthreshold depression. IPC would be useful for implementation in student counseling because it can teach a realistic method of coping with a stressful event and can reduce depression even after just three 50-minute sessions.

## Figures and Tables

**Table 1:** Participant characteristics.

	IPC (*n* = 15)	CAU (*n* = 16)	*p*
Age (years)	20.7 ± 1.4	20.7 ± 0.8	.599
Gender			
Male	3 (20)	3 (19)	
Female	12 (80)	13 (81)	.359
Problems			
Interpersonal relationships	8 (53)	7 (44)	
Studying	3 (20)	5 (31)	
Future career	3 (20)	2 (13)	
Other	1 (7)	2 (13)	.865

*Note. *IPC: interpersonal counseling; CAU: counseling as usual. Continuous variables are expressed as means ± standard deviations. Categorical variables are expressed as frequencies followed by percentages in parentheses. *p* values are derived from Wilcoxon signed-rank test and chi-square tests.

**Table 2:** Outcomes by counseling group.

Outcome measures	IPC	CAU	IPC versus CAU
	(*n* = 15)	(*n* = 16)	**p**
SDS total score			
Precounseling	37.80 ± 8.85	36.19 ± 8.57	.607
Postcounseling	34.53 ± 8.44	36.13 ± 10.02	.607
Pre-post change	−3.27 ± 4.06^*∗*^	−0.06 ± 3.70	.029
CISS score			
Task-oriented coping score			
Precounseling	58.20 ± 10.29	60.69 ± 7.56	.447
Postcounseling	60.40 ± 8.64	59.19 ± 8.89	.429
Pre-post change	2.20 ± 5.39	−1.50 ± 5.34	.065
Emotion-oriented coping score			
Precounseling	41.93 ± 9.13	42.31 ± 10.27	.914
Postcounseling	39.13 ± 11.13	38.88 ± 9.89	.946
Pre-post change	−2.80 ± 6.43	−3.44 ± 7.36	.800
Avoidance-oriented coping score			
Precounseling	44.60 ± 13.09	49.00 ± 11.14	.321
Postcounseling	43.60 ± 12.50	46.13 ± 14.00	.601
Pre-post change	−1.00 ± 7.50	−2.88 ± 6.00	.447

*Note. *IPC: interpersonal counseling; CAU: counseling as usual; SDS: Zung Self-Rating Depression Scale; CISS: Coping Inventory for Stressful Situations. Variables are expressed as means ± standard deviation.  *p* values are derived from Mann–Whitney *U* test for SDS total score at pre- and postcounseling and for task-oriented coping score at postcounseling, while for all other scores *p*values are derived from *t*-tests. Pre-post change within IPC group: * *^*∗*^<.05 derived from Wilcoxon signed-rank test.
